# Improved mapping strategy to better inform policy on the control of schistosomiasis and soil-transmitted helminthiasis in Sierra Leone

**DOI:** 10.1186/1756-3305-4-97

**Published:** 2011-06-06

**Authors:** Mary Hodges, Nsa Dada, Anna Wamsley, Jusufu Paye, Emanuel Nyorkor, Mustapha Sonnie, Guy Barnish, Moses Bockarie, Yaobi Zhang

**Affiliations:** 1 Helen Keller International, Freetown, Sierra Leone; 2 Liverpool School of Tropical Medicine, Liverpool, UK; 3 Faculty of Pure and Applied Science, University of Sierra Leone; 4 School of Community Health Sciences, Njala University, Sierra Leone; 5 Helen Keller International, Regional Office for Africa, Dakar, Senegal

## Abstract

**Background:**

Schistosomiasis and soil-transmitted helminthiasis (STH) are endemic in Sierra Leone confirmed by national mapping in 2008. To better inform planning of preventive chemotherapy strategy, another survey was conducted before mass drug administration (MDA) in seven districts according to the mapping results or local knowledge. Fifty-nine chiefdoms and one school in every chiefdom were selected. Thirty school children aged 9-14 years from each school (total: 1760) were examined by parasitological methods for infection with *Schistosoma mansoni *and STHs.

**Results:**

The overall prevalence of *S. mansoni *was 40.2% (95% confidence interval (CI): 37.9-42.5%), particularly in Kailahun (63.3%), Kenema (46.7%), Koinadugu (41.9%) and Kono (71.7%). The results demonstrated the focal distribution of *S. mansoni *in Bo, Tonkolili and Bombali districts with prevalence ranging from 0.0-63.3%, 3.3-90.0% and 0.0-67.9% respectively. The arithmetic mean intensity of *S. mansoni *infection was 95.4 epg (95% CI: 61.4-129.5 epg), Heavy mean intensity of infection was found in Kailahun (120.2 epg), Kenema (104.5 epg), Koinadugu (112.3 epg) and Kono (250.3 epg). Heavy or moderate infection with *S. mansoni *occurred in 20.7% of children examined. Hookworm prevalence was moderate: 31.2% (95% CI: 29.1-33.4%), but high in Bo (50.0%) and Tonkolili (56.7%). Hookworm intensity of infection was light with a mean epg of 53.0 (95% CI: 38.4-67.7 epg). Prevalence and intensity of *Ascaris lumbricoides *(1.5%, 17.8 epg) and *Trichuris trichiura *(2.5%, 20.3 epg) was low.

**Conclusions:**

The prediction by previous spatial analysis that *S. mansoni *was highly endemic across north-eastern Sierra Leone was confirmed with a significant proportion of children heavily or moderately infected. The distribution of *S. mansoni *in Bo, Tonkolili and Bombali districts ranged widely, highlighting the importance of considering the nature of focal transmission in national mapping exercises. These results were used to refine the MDA for schistosomiasis control to chiefdom implementation units rather than the entire district in these 3 districts. The survey demonstrated that sufficient number of survey sites for schistosomiasis mapping in each district should be used to provide a better national planning of MDA activities, and that it is affordable with the contributions from all parties involved and national resources mobilized.

## Background

Schistosomiasis and soil-transmitted helminthiasis (STH) inflict a significant health and socioeconomic burden on the poorest populations in rural and poor urban settings in the tropics and subtropics, in particular, in sub-Saharan Africa [1-3]. It is estimated that over 200 million people are infected with schistosomes worldwide and about two billion with STHs [2,3]. Schistosomiasis, caused by *Schistosoma mansoni *or *S. haematobium*, and STH, of particular worldwide importance, caused by *Ascaris lumbricoides*, *Trichuris trichiura*, and hookworms (*Ancylostoma duodenale *and *Necator americanus*), cause significant morbidity worldwide with a combined 43.5 million or more disability adjusted life years (DALYs) lost each year, more than those lost to malaria (36 million) and approaching those lost to tuberculosis (47 million) [4-6]. Schistosomiasis alone could be responsible for 200 000 deaths per year in sub-Saharan Africa, and STH could be responsible for 135 000 deaths per year globally [7].

School-aged children, young adolescents and pre-school children tend to harbour the greatest number of worms, and as a result experience stunting, wasting, diminished physical fitness as well as impaired memory and cognitive abilities [5,8]. These adverse health consequences combine to impair childhood educational performance and reduce school attendance, leading to a reduction in future productivity and wage-earning capacity [5,9]. These infections also have direct and indirect effects on malaria and HIV/AIDS in developing countries where they are co-endemic [10].

Control of schistosomiasis and STH is now part of the global effort in the integrated control of the neglected tropical diseases (NTDs) which currently targets five major 'tool-ready' diseases (lymphatic filariasis, onchocerciasis, schistosomiasis, STH and trachoma) through preventive chemotherapy [11]. The drugs needed for these five NTDs are robust, safe, low-cost and available by donation from the pharmaceutical companies or by purchasing at relatively low costs. The funds are provided by a number of governmental and non-governmental organisations, notably, the United States Agency for International Development, British Government Department for International Development, and the Bill & Melinda Gates Foundation. This drug-based intervention delivers available drugs either alone or in combination to prevent morbidity caused by these NTDs, or in some cases to eliminate the diseases. There is currently a major drive from the World Health Organization (WHO) to prepare the countries in sub-Saharan Africa for such national NTD control programmes. Geographical mapping of these NTDs is required as an essential tool for directing national intervention programmes.

Both intestinal and urinary forms of schistosomiasis are known to be endemic in Sierra Leone [12-16], as well as STHs [17-21]. Various prevalence data on STHs in school aged children are available from the 1990s, with *A. lumbricoides *32-93%, hookworm 25-43%, and *T. trichiura *39-75% [22-25]. National mapping of the prevalence and distribution of intestinal schistosomiasis and STH was conducted in Sierra Leone in 2008 [26]. In the 2008 mapping survey, four survey sites (schools) per district were selected according to administrative districts using a two-staged random sampling method to avoid two schools being selected from the same chiefdom to ensure a relatively even geographical coverage throughout the country as described previously [26]. Spatial analysis predicted that *S. mansoni *was highly prevalent in Sierra Leone with a large cluster of high risk of *S. mansoni *infection (prevalence >70%) in the northeast half of the country where annual preventive chemotherapy (PCT) intervention was required and that hookworm infection was high across the country with a large cluster of high infection risk (prevalence >70%) in the north-eastern part [26]. However, it was noted that the relatively small number of randomly sampled sites (only four per district) used in the mapping might have underestimated the endemicity, particularly of schistosomiasis in the Bo and possibly Bombali districts. Therefore, this complementary survey was conducted in 2009 in seven districts before the mass drug administration (MDA) was implemented.

The objective of the current survey was (1) to validate the predicted prevalence maps of *S. mansoni *and STHs in districts where the mapping suggested that widespread MDA was needed and in particular in Bo district where local knowledge suggested that schistosomiasis was indeed endemic, (2) to refine the MDA strategy in these districts in the integrated national NTD control programme, and (3) to provide intensity of infection data for *S. mansoni *and STHs among school aged children in the endemic areas, which was not reported earlier.

## Methods

### Sampling and data collection

The survey was carried out in 2009 in seven rural districts, Bo, Bombali, Kenema, Koinadugu, Kailahun, Kono and Tonkolili. These districts were shown to be highly endemic across the districts with *S. mansoni *according to the 2008 survey and spatial prediction, except for Bo where local knowledge suggests that it is also endemic for schistosomiasis but the 2008 mapping had failed to identify this. Fifty-nine (59) chiefdoms in these seven districts (9 chiefdoms per district from Bo, Bombali, Kailahun and Kenema, 8 chiefdoms per district from Kono and Tonkolili, and 7 chiefdoms from Koinadugu) which were not sampled in the mapping in 2008 but were predicted to be highly prevalent with *S. mansoni *were selected. One primary school per chiefdom was sampled based on accessibility. Within each school, around 30 children aged 9-14 years who volunteered to participate were enrolled, balancing for sex.

During sensitisation by students attending the University of Sierra Leone (USL) and Njala University (NU), all participants were provided with leak-proof specimen container, and instructions. The fresh stool samples were collected within a few hours, flooded with 10% formalin, shaken to arrest development of ova or larvae and an identification number for each sample was allocated. Samples were transported back and examined in laboratories at USL and NU. One slide per stool sample was prepared and examined by experienced parasitological examiners with the Kato-Katz thick smear technique using a 41.7 mg template. Number of parasite eggs per slide was recorded and intensity of infection was calculated and expressed as eggs per gram (epg) of faeces. Individual infections with *S. mansoni *and STHs were classified as light, moderate or heavy according to the WHO classifications [27].

### Data analysis

Results obtained were entered into computer using Epi info^® ^software (version 3.5.1) and exported into SPSS (IBM, version 18) for analysis. The size of districts and the number of schools existing within each district varied significantly; therefore, sample weighting was applied during calculation. Sample weights were calculated for each district according to the ratio of the proportionally expected number of schools to be surveyed (total number of schools within a district × overall proportion of schools selected in seven districts) and the number of actually surveyed schools in each district. Prevalence and arithmetic mean intensity of infection among all children examined with 95% confidence intervals (CIs) were obtained using the Complex Samples analysis in the SPSS, stratified by districts. The 95% CIs for prevalence were calculated using the Wilson score method without continuity correction after adjusting for sample weighting [28]. Differences in prevalence were compared using Chi-square test, and differences in intensity of infection were compared using Mann-Whitney test for two samples and Kruskal-Wallis test for multi groups. The coordinates of each sample site were documented using hand-held units of global positioning system. The prevalence maps of geographical distribution of each disease using observed point prevalence from each school were produced using ArcMap software (ESRI, version 9.3).

Ethical approval for the survey was obtained from the Ministry of Health and Sanitation, Sierra Leone and the Liverpool School of Tropical Medicine Research Ethics Committee. Community informed consent was obtained following discussion with District Medical Officers, Chiefdom school inspectors, head teachers and community-teachers associations. Verbal individual consent was obtained from parents/carers as literacy rates among parents are low in these locations.

## Results

### Overall prevalence and intensity of infection

A total of 1,760 faecal specimens were examined with two samples missing age and sex data, (males 51.4% and females 48.6%). The mean age (± standard deviation) in male was 11.52 ± 1.67 and in female 11.30 ± 1.68.

Table 1 summarizes the overall prevalence and intensity of infection in the children examined. Overall *S. mansoni *prevalence was moderate: 40.2% (95% CI: 37.9-42.5%) and also for hookworm: 31.2% (95% CI: 29.1-33.4%). Overall prevalence for *A. lumbricoides *was low: 1.5% (95% CI: 1.1-2.2%) and for *T. trichiura: *2.5% (95% CI: 1.9-3.4%). There were 33.0% (95% CI: 30.8-35.2%) of children infected with any STHs, and 60.7% (95% CI: 58.4-63.0%) of children infected with any of four parasites surveyed. There were no significant differences between sex in *S. mansoni*, *A. lumbricoides *or *T. trichiura *infection (p > 0.05), but significantly more boys were infected with hookworms than girls (p = 0.002). There was no difference between age groups in any of four infections (p > 0.05).

**Table 1:** Adjusted *S. mansoni *and STH prevalence and intensity of infection (95% CI) in 9-14 years old in seven districts, Sierra Leone

	No of subjects	*S. mansoni *	*Hookworm *	*A. lumbricoides*	*T. trichiura*
**Prevalence (%)**					
Overall prevalence	1760	40.2 (37.9 - 42.5)	31.2 (29.1 - 33.4)	1.5 (1.1 - 2.2)	2.5 (1.9 - 3.4)
Sex					
Boys	904	41.7 (38.5 - 45.0)	35.3 (32.3 - 38.5)	1.3 (0.8 - 2.3)	2.2 (1.4 - 3.4)
Girls	854	38.5 (35.3 - 41.8)	27.0 (24.1 - 30.1)	1.8 (1.1 - 2.9)	2.9 (2.0 - 4.3)
					
**Intensity of Infection (epg)**					
Overall mean epg	1760	95.4 (61.4 - 129.5)	53.0 (38.4 - 67.7)	17.8 (0.0 - 38.9)	20.3 (0.0 - 48.5)
Sex					
Boys	904	96.6 (64.2 - 129.0)	60.2 (43.9 - 76.5)	11.8 (1.0 - 22.7)	12.2 (0.0 - 27.5)
Girls	854	94.2 (56.0 - 132.4)	45.7 (24.3 - 67.2)	24.1 (0.0 - 66.5)	28.9 (0.0 - 83.8)

Overall intensity of *S. mansoni *infection was 95.4 epg (95% CI: 61.4-129.5 epg); hookworm: 53.0 epg (95% CI: 38.4-67.7 epg); *A. lumbricoides*: 17.8 epg (95% CI: 0.0-38.9 epg); and *T. trichiura*: 20.3 epg (95% CI: 0.0-48.5 epg). There was no significant difference in intensity of infection between sex or between ages in *S. mansoni*, *A. lumbricoides *or *T. trichiura *infection (p > 0.05), but boys were more heavily infected with hookworms than girls (p < 0.01). Based on the WHO classifications [27], there were 20.7% of children heavily or moderately infected with *S. mansoni*, while all infections with STHs were relatively light (details not shown).

### Geographical distribution

**Figure 1:**
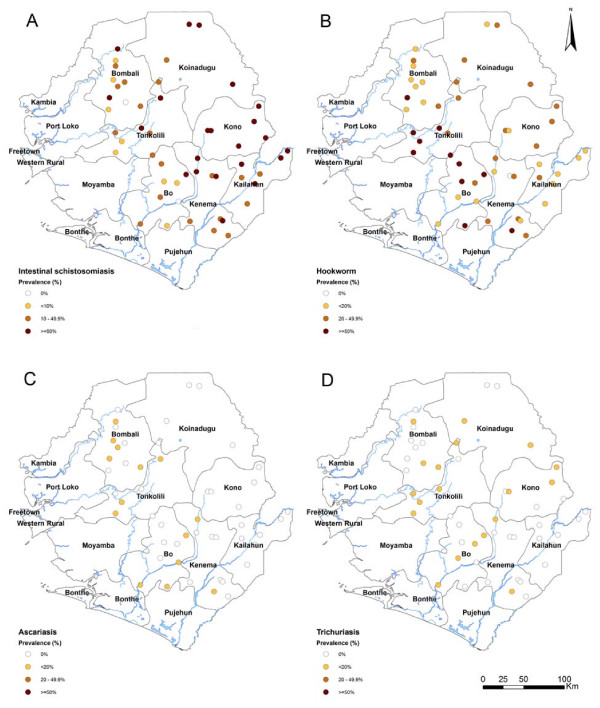
**Geographical distribution of intestinal schistosomiasis and soil-transmitted helminthiasis in selected schools in seven districts, Sierra Leone**. Observed point prevalence is shown by locations of the selected schools: (A) *S. mansoni*, (B) Hookworm, (C) *A. lumbricoides*, and (D) *T. trichiura*.

Table 2 summarizes the prevalence and intensity of infection of *S. mansoni *and hookworm by district as these were the main parasites confirmed (data for *A. lumbricoides *and *T. trichiura *not shown due to low levels of infection). Figure 1 shows the point prevalence distribution of *S. mansoni *and STHs in the schools surveyed in the seven districts. *S. mansoni *infection was confirmed in all seven districts with high level of infection in the most north-easterly districts: Kono (median prevalence: 71.7%; arithmetic mean intensity of infection: 250.3 epg), Kailahun (63.3%; 120.2 epg), Kenema (46.7%; 104.5 epg) and Koinadugu (41.9%; 112.3 epg).

**Table 2:** *S. mansoni *and hookworm median prevalence (inter-quantile range, minimum-maximum range) and arithmetic mean intensity of infection (95% CI) by districts, Sierra Leone

	No of subjects	*S. mansoni*	*Hookworm*
**Prevalence (%)**			
Bo	269	6.7 (3.3-13.3, 0.0-63.3)	50.0 (17.2-53.3, 6.7-66.7)
Bombali	261	13.3 (3.3-29.6, 0.0-67.9)	11.1 (10.0-17.9, 3.4-46.7)
Kailahun	270	63.3 (43.3-66.7, 43.3-73.3)	16.7 (10.0-20.0, 3.3-23.3)
Kenema	269	46.7 (23.3-50.0, 17.2-96.7)	26.7 (16.7-36.7, 0.0-60.0)
Koinadugu	211	41.9 (30.0-83.3, 13.3-93.3)	26.7 (16.7-35.0, 9.7-46.7)
Kono	240	71.7 (58.3-82.5, 53.3-93.3)	31.7 (22.5-36.7, 10.0-40.0)
Tonkolili	240	18.3 (14.2-74.2, 3.3-90.0)	56.7 (50.0-57.5, 26.7-66.7)
			
**Intensity of Infection (epg)**			
Bo	269	17.8 (1.9-33.8)	68.2 (36.5-99.9)
Bombali	261	32.1 (1.6-62.6)	12.5 (0.0-28.6)
Kailahun	270	120.2 (31.5-208.8)	7.7 (2.8-12.6)
Kenema	269	104.5 (0.0-210.8)	38.8 (13.3-64.3)
Koinadugu	211	112.3 (26.7-197.9)	25.7 (15.3-36.1)
Kono	240	250.3 (95.0-405.6)	29.9 (17.5-42.3)
Tonkolili	240	80.5 (9.2-151.8)	139.9 (72.0-207.8)

In Bo district where the 2008 mapping survey did not find *S. mansoni *infection it was confirmed in eight out of nine survey sites with prevalence ranging 3.3-63.3% and a mean intensity of 17.8 epg (95% CI: 1.9-33.8). The focal distribution of *S. mansoni *was also confirmed in Tonkolili and Bombali with prevalence ranging from 3-90% and 0%-67.9% respectively and mean intensity of 32.1 epg (95% CI: 1.6-62.6) and 80.5 epg (95% CI: 9.2-151.8) respectively.

Hookworm prevalence was high in Tonkolili (56.7%; 139.9 epg) and Bo (50%; 68.2 epg), moderate in Kailahun (16.7%, 7.7 epg), Kenema (26.7%, 38.8 epg), Kono (31.7%, 29.9 epg) and Koinadugu (26.7%, 25.7 epg) and low in Bombali (11.1%, 12.5 epg).

### Costs of the survey

The field work was performed with the support of undergraduate students from the University of Sierra Leone and Njala University and laboratory work with support from Liverpool School of Tropical Medicine. The total expenditure on the survey, including travel, field work allowances, laboratory examinations etc was 18,375 US dollars, an equivalent of $311 per site surveyed. This does not include the personnel salaries/wages.

## Discussion

Planning and implementation of preventive chemotherapy strategy recommended by the WHO depends on a good understanding of endemic situation of the diseases in each district [29-31]. This is particularly the case for schistosomiasis because of its focal distribution in areas, close to snail-infected water sources which in Sierra Leone are altitude related [12]. The mapping survey conducted in 2008, prior to the MDA of the national integrated NTD control programme, gave an overall picture of *S. mansoni *and STH endemic situations in Sierra Leone [26]. Together with spatial prediction, the northeast half of the country was classified as highly endemic for *S. mansoni*, which required annual MDA for all school-age children plus adults at high risk. However, in the 2008 survey relatively few (four) sites per district were used, therefore there was a need to validate the predicted prevalence maps and confirm the PCT strategy in chiefdoms that were not surveyed. Furthermore, in three districts (Bo, Bombali and Tonkolili) it was unclear from the 2008 mapping whether MDA was justified and if so how frequently it should be performed.

The results of this current survey confirmed the high endemicity of *S. mansoni *in Kailahun, Kenema, Koinadugu and Kono districts and in Eastern chiefdoms of Bombali and Tonkolili districts, suggesting a convincing prediction by the spatial analysis in these areas. The results also confirmed that some chiefdoms in Bo district were indeed endemic and therefore qualified for MDA. This highlights the importance of sampling methods in schistosomiasis survey to meet the nature of focal transmission of the disease.

The results also provided valuable data on the intensity of infection of *S. mansoni *and STHs in school aged children in these areas which were not recorded in the 2008 mapping survey. School aged children in Kailahun, Kenema, Koinadugu and Kono districts are relatively heavily infected with *S. mansoni *with arithmetic mean intensities of infection of over 100 epg. There are 20.7% of children heavily or moderately infected with *S. mansoni *in these seven districts. This highlights the need for intervention to prevent severe morbidity in their later life [32]. Based on these survey data, MDA was planned and these highly endemic districts have so far received two rounds of treatment In 2009 school-going children were targeted, and 562,980 received praziquantel and 549,701 received mebendazole. In 2010, all school-aged children and at risk adults were targeted, and 1,826,284 received praziquantel and 1,000,042 received albendazole [33].

The focal distribution of *S. mansoni *is most clearly demonstrated in Bo, Bombali and Tonkolili with prevalence widely ranging from chiefdom to chiefdom. In the context of such districts in Sierra Leone, it is important to plan the implementation strategy according to the chiefdoms rather than the whole districts. This complementary survey provided such a tool to enable the national program managers and district health teams to plan and refine their MDA activities in conjunction with the 2008 mapping results. This is a clear demonstration that a detailed mapping survey with sufficient number of survey sites across the endemic areas, in particular for focal diseases such as schistosomiasis, is needed in order to better plan the control activities. There is a dilemma currently for the national teams in terms of the need of detailed mapping and the funds available. The current survey of about $310 per site also demonstrated the affordability of such mapping surveys with the contributions from all parties involved and national resources mobilized.

Low prevalence and intensity of *A. lumbricoides *and *T. trichiura *and moderate prevalence and light intensity with hookworm highlight remarkable reductions in comparison with pre-war studies [18-20,23,24]. The impact on STH of the annual distribution of ivermectin and albendazole in the NTDCP's lymphatic filariasis elimination programme since 2007 and various school-based deworming programs performed by the Wood Food Program and NGOs since 2004 has been discussed previously [26,33]. Although of light intensity, the moderate prevalence of hookworm infections and widespread anaemia in children in Sierra Leone according to the national data confirm the desirability of using albendazole as the drug of choice as albendazole has a better cure rate for hookworm infections [34].

The current survey provided a significantly improved tool for planning the MDA activities in each district, however there are certain limitations in the methodology used. Firstly, the sampling method of survey sites based upon accessibility, though not uncommon for schistosomiasis, may not represent the true endemicity level in chiefdoms, as those schools in hard-to-reach locations may be heavily affected by schistosomiasis. Secondly, by adding formalin to the stool samples, it may have diluted the stool samples and caused underestimation of the results. However, considering the rural conditions in the post-war Sierra Leone, it was not possible to process and examine the stool samples on site, and therefore, preservation of the samples was a necessary trade-off to avoid disintegration of helminth, particularly hookworm, eggs. Thirdly, only one Kato-Katz slide was used for the diagnosis, and the low sensitivity of this method may have caused underestimation of the prevalence and slight overestimation of the intensity of infection. However, considering the morbidity control strategy for schistosomiasis and STH by preventive chemotherapy in the current national control programme, such slight misestimation would not make much difference to the overall objectives of the national programme.

## Conclusions

The current complementary survey confirmed that *S. mansoni *is indeed endemic across the northeast half of Sierra Leone, particularly in Kailahun, Kenema, Koinadugu, Kono, North-eastern Tonkolili and Eastern Bombali. It also confirmed that *S. mansoni *is prevalent in Bo district as the local knowledge anticipated, highlighting the importance of sampling survey sites for schistosomiasis survey according to the local knowledge and environmental situations. There are over 20% of children heavily or moderately infected with *S. mansoni *in these seven districts, justifying the need for MDA to prevent severe morbidity in their later life. This study clarified that MDA was justified annually throughout Kailahun, Kenema, Koinadugu, Kono, North-eastern Tonkolili, Eastern Bombali and in certain chiefdoms in Bo. The survey demonstrated that sufficient number of survey sites for schistosomiasis mapping in each district should be used to provide a better national planning of MDA activities.

## Competing interests

The authors declare that they have no competing interests.

## Authors' contributions

MH conceived, designed, supervised the study and revised the paper; JP, EN and MS coordinated the field data collection; ND, AW, JP performed the laboratory investigations, ND and AW conducted data entry and initial data analysis; YZ conducted final data analysis using SPSS and ArcMap, and MH and YZ drafted and revised the paper. GB, MB supervised ND and AW and reviewed the paper. All authors reviewed and approved the final manuscript.
